# Hypo-activity of the dorsolateral prefrontal cortex relates to increased reaction time variability in patients with schizophrenia

**DOI:** 10.1016/j.nicl.2019.101853

**Published:** 2019-05-03

**Authors:** G. Panagiotaropoulou, E. Thrapsanioti, E. Pappa, C. Grigoras, D. Mylonas, E. Karavasilis, G. Velonakis, N. Kelekis, N. Smyrnis

**Affiliations:** a Department of Psychiatry, National and Kapodistrian University of Athens, School of Medicine, Eginition Hospital, Athens, Greece; b Laboratory of Cognitive Neuroscience and Sensorimotor Control, University Mental Health, Neurosciences and Precision Medicine Research Institute “COSTAS STEFANIS”, Athens, Greece; c Second Department of Radiology, National and Kapodistrian University of Athens, School of Medicine, University General Hospital ‘Attikon’, Athens, Greece; d MGH/HST Athinoula A. Martinos Center for Biomedical Imaging, Massachusetts General Hospital, Boston, MA, USA; e Department of Psychiatry, Harvard Medical School, Boston, MA, USA

**Keywords:** fMRI, Attention, Prefrontal cortex, Reaction time, Intra-subject variability, Psychosis

## Abstract

Increased reaction time intra-subject variability (RT-ISV) in fast decision tasks has been confirmed in patients with schizophrenia and has been hypothesized to result from a deficit in the control of attention. Here, an attentional task and functional brain imaging were used to probe the neural correlates of increased RT-ISV in schizophrenia.

Thirty patients and 30 age and sex matched controls performed the Eriksen flanker spatial attention task with concurrent measurement of brain activity using functional magnetic resonance imaging (fMRI). The behavioral measures included accuracy, mean, standard deviation of RT (RTSD), coefficient of variation of RT (RTCV) and ex-Gaussian model of RT distribution parameters (mu, sigma and tau).

Larger mean RT and Ex-Gaussian mu was observed for patients compared to controls. The group difference was larger for incongruent (attentionally demanding) versus congruent trials confirming a deficit in the control of spatial attention for patients. Significant increase in RT-ISV measures (RTSD, sigma and tau) for patients compared to controls was observed and was not modulated by trial congruency. Attention modulation (congruency effect) resulted in activation of bilateral frontal and parietal areas that was not different between patients and controls. Right middle frontal, right superior temporal and bilateral cingulate areas were more active in controls compared to patients independent of congruency. Activation in ROIs extracted from attention (congruency) and group related areas correlated with RT-ISV measures (especially RTCV and tau). Hypo-activation of the right middle frontal area correlated with increased tau specifically in patients.

Hypo-activity of the right prefrontal cortex predicted increased RT-ISV in schizophrenia. This effect was unrelated to the effects of spatial attention and might be linked to a deficit in the inhibitory control of action for these patients.

## Highlights

• Schizophrenia patients show increased reaction time intra-subject variability (RT-ISV).
• fMRI of patients and aged matched controls while performing a spatial attention task.
• RT-ISV measures were increased in patients independent of spatial attention load.
• Activity of the right middle frontal cortex predicted RT-ISV increase in patients.

## 1 Introduction

Studies of decision processing in simple sensorimotor tasks have confirmed that patients with schizophrenia have higher mean reaction time (RT) compared to controls (Cadenhead et al., 1997; Nuechterlein, 1977; Shakow, 1962). The distribution of RT in such simple sensorimotor decision tasks carries more information than can be captured by mean RT. One such piece of information is the variance of the RT distribution, which has been termed RT intra-subject variability (RT-ISV). RT-ISV has been proposed as a specific measure of cognitive and sensorimotor processing stability that is independent of the mean RT (Kuntsi and Klein, 2011; Rentrop et al., 2010). Patients with schizophrenia showed increased RT-ISV in sensorimotor decision tasks compared to healthy controls (Nuechterlein, 1977; Shakow, 1962). Increased RT-ISV has been extensively documented as a behavioral biomarker in children with Attention Deficit Hyperactivity Disorder (ADHD) (Johnson et al., 2007; Klein et al., 2006). This literature favored the hypothesis that RT-ISV is related to a deficit in the control of attention leading to attentional lapses that are reflected in the large variation of RT from trial to trial in children with ADHD (Tamm et al., 2012).

Recent studies have examined RT-ISV as a biomarker for schizophrenia. For instance, mean RT was larger for all groups with psychotic symptoms (schizophrenia and affective disorders), whereas RT-ISV was larger specifically for schizophrenia patients (Schwartz et al., 1989). Another study revealed a dissociation of mean RT and RT-ISV in schizophrenia, the first specifically predicting the inability of patients to maintain a cognitive set, the second specifically predicting the severity of psychotic and disorganization symptoms (Vinogradov et al., 1998). Comparing RT measures among schizophrenia, major depression and borderline personality disorder patients, increased RT-ISV dissociated schizophrenia patients from all other groups (Kaiser et al., 2008). Higher RT-ISV, but not mean RT, was associated with impaired performance in patients with schizophrenia and major depression (Kaiser et al., 2008; Van den Bosch et al., 1996). In a visually guided saccade task, RT-ISV but not median RT, was larger in patients with schizophrenia compared to healthy controls (Smyrnis et al., 2009). An increase in the variation of the decision signal leading to saccade (corresponding to a measure of RT-ISV) dissociated patients with schizophrenia from healthy controls and patients with obsessive compulsive disorder (Theleritis et al., 2014). Finally, increased RT-ISV measures dissociated patients with schizophrenia and their first-degree relatives from healthy controls in a working memory task (Fish et al., 2018).

The standard measures of RT distribution in the study of sensorimotor decision tasks in schizophrenia have been the mean (or median) RT and as measures of RT-ISV, the standard deviation of RT (RTSD) and the coefficient of variation (RTCV) (which is the ratio of standard deviation to the mean RT). The basic assumption for using these measures is that the RT distribution can be approximated by the Gaussian distribution. This assumption though is not true and it is clearly shown that the RT distribution deviates from normality and is heavily skewed to the right (Luce 1986). In an attempt to capture the shape of the RT distribution other models were used such as the log-normal (Luce 1986), the reciprocal of RT (Saville et al., 2011) and the ex-Gaussian (Heathcote et al., 1991; Hohle, 1965; Luce, 1986; Ratcliff and Murdock, 1976; Saville et al., 2011). The ex-Gaussian model uses the combination of a Gaussian component and an exponential component and provides three basic parameters: mu (*μ*) and sigma (*σ*) correspond to the mean and SD of the Gaussian component, while tau (*τ*) models the slope of the exponential component corresponding to the long tail of the RT distribution. While it was initially suggested that tau measures the decision component of the underlying cognitive process while mu and sigma measure the non-decisional sensorimotor processes (Hohle, 1965), more recent work has shown that the relation of the ex-Gaussian parameters to the underlying cognitive processes is more complex (Matzke and Wagenmakers, 2009) and the ex-Gaussian parameters should be viewed as descriptive of the RT distribution (Heathcote et al., 1991).

The ex-Gaussian model has been extensively used in the study of RT-ISV in children with ADHD and a specific increase in tau has been proposed as a behavioral biomarker of the disorder (Lin et al., 2015). A small number of studies employing different cognitive tasks have used the ex-Gaussian model to investigate RT-ISV in schizophrenia. In one study mu and tau, but not sigma, were increased in patients compared to controls (Kieffaber et al., 2006), while in a second study only tau was significantly increased in schizophrenia patients compared to controls (Rentrop et al., 2010). A significant increase in sigma and tau, but not mu, was observed in a simple saccade and manual RT task (Karantinos et al., 2014). Finally, findings from a recent study using a working memory task showed an increase in all three ex-Gaussian components, dissociating patients with schizophrenia and their first-degree relatives from healthy controls (Fish et al., 2018). These studies then give rise to the hypothesis that, in analogy to ADHD, RT-ISV and especially the long tail of the RT distribution modelled by ex-Gaussian tau might also serve as a biomarker for schizophrenia. This hypothesis in turn raises new questions. Is the RT-ISV increase in schizophrenia related to a deficit in the executive control of attention as has been hypothesized for ADHD (Tamm et al., 2012)? What are the neural correlates of the increase in RT-ISV in schizophrenia?

To our knowledge, the only study that has so far investigated the neural correlates of increased RT-ISV in schizophrenia used the Stroop task of executive control of attention (Fassbender et al., 2014). In the analysis of the imaging data, RT was divided into bins and was used as a predictor of neural activity in both controls and patients. Several areas including the dorsolateral prefrontal cortex, posterior parietal cortex and middle cingulate cortex were more active in controls compared to patients. This increased activation was specific for large RTs. These differences though do not directly relate to RT-ISV measures.

The present study aimed to specifically explore the neural correlates of increased RT-ISV in schizophrenia. Healthy controls and patients with schizophrenia performed the “arrow head” spatial version of the Eriksen flanker spatial attention task (Davelaar and Stevens, 2009; Egner, 2007; Gratton et al., 1992; Nieuwenhuis et al., 2006) while measuring whole brain activity with fMRI. The choice of an attention task that also involves fast decision processing allowed us to investigate whether activation in specific attention related brain areas would correlate with measures of RT distribution and whether activation differences in these or other brain areas could predict differences in RT-ISV measures between patients and controls.

## 2 Material and methods

### 2.1 Participants

Our sample consisted of 30 patients with DSM-IV schizophrenia (mean age: 29.6y, SD: 7.7y, 25 men, 5 women) and 30 healthy controls (mean age: 27.8y, SD: 7.7y, 23 men, 7 women). Groups were matched for age (t_58_ = 0.88, *p* = .38) and sex (X^2^_1_ = 0.42, *p* = .52). The study protocol was approved by the Ethics committee of Eginition University Hospital. Participants provided written informed consent. Patients were hospitalized in the psychosis inpatient unit of Eginition University Hospital and their diagnosis was confirmed by a trained psychiatrist using the DIP-DM diagnostic module (McGuffin et al., 1991). Mean duration of psychosis was 8.5y (SD = 6.6y). Patient participants were treated with antipsychotic medications during testing (chlorpromazine equivalent dose, mean: 527.3 SD: 348.9) and were in a remission phase of the disorder a few days prior to, or after discharge from the inpatient unit (Kroken et al., 2009). In addition to antipsychotic medication, 7 patients received antidepressant medication and 2 of them received also a mood stabilizer. Patients were not receiving benzodiazepines and/or b-blockers. Exclusion criteria were organic cerebral illness, mental retardation and other major psychiatric disorder comorbidity. Systematic cannabis and other illicit drug abuse just prior to admission to the inpatient unit was also a criterion for exclusion. Healthy controls were also screened for a history of mental disorders.

### 2.2 Behavioral task design

Participants performed the arrow head version of the Eriksen flanker task, using two MR compatible response pads (CEDRUS equipment). The task was programmed using the *E*-Prime (version 2.0) software. In each trial participants fixated on a central stimulus (cross “+”) for a variable period of 2, 4 or 6 s. Then a cue replaced the central fixation stimulus (circle “o”) and remained visible for 0.5 s. After the cue, the response stimulus was presented for 0.5 s. The stimulus included a series of 5 arrow heads, a central one and 4 peripheral arrow heads (flankers) pointing to the same direction as the central one (“>>>>>”, congruent stimulus) or to the opposite direction (“>><>>”, incongruent stimulus). Participants were instructed to respond to the direction of the central arrow head by pressing a button on one of two response pads using the right or left index finger accordingly. The total time allowed for response was 1.5 s (0.5 s stimulus presentation and 1 s till the end of response time). Therefore, the total trial time varied randomly among 4, 6 or 8 s. Each participant performed 75 trials for each combination of responding hand and congruency (right hand congruent, right hand incongruent, left hand congruent, left hand incongruent) for a total of 300 trials. Trials were administered in a random order and were divided in three blocks of 100 trials each. Between blocks participants were given few minutes of rest while remaining inside the scanner. A few trials were also administered prior to scanning in order to familiarize participants with the task procedure.

### 2.3 Behavioral data acquisition and analysis

Reaction Time (RT) and correct responses were measured for each participant and each trial type. Then accuracy (percentage of correct responses), mean reaction time for correct responses (RTM), standard deviation of reaction time for correct responses (RTSD) and the coefficient of variation of reaction time for correct responses (RTCV) (which is the standard deviation divided by the mean) were calculated. One patient performed <10% correct trials during the incongruent task condition, so her data were excluded from further analysis, resulting in valid behavioral data for 29 patients and 30 controls. All other participants had >50% correct answers. There was no significant difference in accuracy, RTM, RTSD or RTCV between right and left hand responses for patients and controls. Thus, right and left hand response data were pooled for each participant for the behavioral as well as the functional imaging data analysis.

The ex-Gaussian model was fit, to each one of the RT distributions of correct congruent and correct incongruent trials for each participant and parameters mu, sigma and tau were derived. The model was implemented with the use of the “egfit” function (Lacouture and Cousineau, 2008) in Matlab (Mathworks, version 2014). To test the robustness of the ex-Gaussian model fit to the RT distributions log-normal distribution model was also fitted to each RT distribution for comparison. The “lognfit” function of Matlab (Mathworks, version 2014) was used for this fit. For each RT distribution, for each subject and trial type (congruent, incongruent), we compared the goodness of fit between the ex-Gaussian and the lognormal model using Akaike's Information Criterion (AIC). The comparison favored the ex-Gaussian model for 96 RT distributions (81%) that had a smaller AIC value and the log-normal model for 22 RT distributions (19%). Also, visual inspection of the Ex-Gaussian fit to each RT distribution showed excellent fit in all cases. This analysis justified the selection of the ex-Gaussian model for modeling RT distributions in our sample.

The group and congruency effects were tested on behavioral measures using a repeated measure ANOVA. Congruency (congruent and incongruent stimulus trials) was the within-subject repeated measures factor, while group membership (patients and control subjects) was the across-subject factor. For the patient group, correlation between all behavioral measures and duration of psychosis as well as antipsychotic medication dose was performed.

### 2.4 Functional imaging data acquisition

Functional MR images were acquired with a Philips Achieva 3.0 Tesla TX MRI scanner equipped with echo-planar imaging (TR = 2 s, 36 slices covering almost all of the cerebral cortex, voxel size 3 × 3 × 3). An anatomical image of high resolution (T1 image, voxel size 1 × 1 × 1) was acquired for each participant.

The SPM12 Toolbox (Wellcome Trust Centre for Neuroimaging, London, UK) for MATLAB was used to preprocess the data and perform standard general linear model (GLM) analysis. Raw images were spatially realigned (motion correction) and temporally interpolated to compensate for acquisition delay. Subjects with registered motion over 1 mm or 1 degree were discarded. Also, a *t*-test was performed between the motion parameters of the two groups (patients and controls), to ensure that no differential motion was introduced. The high-resolution anatomical image was used to perform tissue segmentation into gray and white matter and cerebrospinal fluid (CSF). Images were next normalized to standard MNI space and smoothed with an 8 mm FWHM (full width at half maximum) Gaussian kernel. Following the preprocessing stage, high-pass filtering of 128 s cut-off was applied to the voxel time-series to remove low-pass physiological components such as respiration and heartbeat. Imaging data from one patient were corrupted by increased levels of artifact due to increased head motion during imaging so the final imaging analysis was performed for 28 patients and 30 controls.

### 2.5 Whole-brain analysis

A first-level (subject-specific) analysis was performed using SPM, whereby a General Linear Model (GLM) was fitted to the imaging data from all 3 sessions acquired from each participant. Following an event-related design, the event onsets of the four conditions (Congruent Left, Congruent Right, Incongruent Left, Incongruent Right) of our experiment were grouped into two (Congruent and Incongruent) and used as independent regressors in the design matrix following convolution with the canonical hemodynamic response function (HRF). The use of temporal and dispersion derivatives was not considered a necessity on the basis that an event-related design was used and that reaction times for this particular task did not exceed 2 s. HRF basis modeling was opted, in order to take advantage of the directionality of t-contrasts (see group-level analysis), while avoiding bias on the parameter estimates that is usually introduced by the presence of derivatives in the model estimation, resulting in artificially low HRF estimates by attributing variance to the derivatives (Lindquist et al., 2009). Nuisance covariates estimated from the motion correction step were also included as additional regressors along with a constant column for each session, modeling the baseline voxel activation. T-contrasts were employed to assess the effect of each condition of interest (congruent or incongruent) against the baseline as well as the positive effect of each condition against the other (incongruent – congruent) and (congruent – incongruent).

A second-level (group-level) analysis was subsequently performed on the contrasts of interest from each group of subjects, namely controls and patients. A factorial design was employed with *group* (control or patient) as the outer two-level factor and *congruency* (congruent or incongruent) as a nested repeated measures factor. For each participant the t-contrast statistical maps produced by the SPM software. The effect of each task condition against the baseline was used as input to the second-level model.

### 2.6 Region of interest (ROI) analysis

In order to investigate the relation of task- and group-specific activated regions to the measures of RT distribution, a region of interest (ROI) analysis was employed. Using MarsBar group-level regions of interest were defined as 10 mm-radius spheres around the highest voxel peaks of each statistically significant cluster from both the (incongruent – congruent) and the (controls – patients) contrasts (trial-type specific or task-specific ROIs and group-specific ROIs respectively). When a cluster included more than one distinct peak, separate ROIs for all peaks were created. The group ROIs were then structurally confined to lie entirely within the corresponding gyrus using the anatomical regions provided by the AAL atlas. In order to account for inter-subject structural and functional variability, we subsequently defined individualized ROIs for each subject by locating the subject-specific peak of activation within each one of the group ROIs and then defining an individual ROI of 3 mm radius around this peak. Task-specific and group specific ROIs can be seen in Fig. 2, Fig. 3 respectively. Using MarsBar, the average time-series for each ROI for each subject was extracted. The effect size of each of the congruent and incongruent conditions was calculated. The conditions are orthogonal, thus the statistical effect sizes provide a good proxy for the task-related ROI activation, namely the change in the BOLD signal during each of these conditions compared to the baseline.

RT measures (RTM, RTSD, mu, sigma and tau) and task related ROI activations (measured by ROI effect size) where standardized so as to be comparable between models. In order to avoid correlation between regressors, ROI activations where additionally orthogonalized with respect to group and trial type and each one was used to fit a linear mixed model to each one of the RT measures using ROI, group and trial-type as well as their two-way and three-way interactions as fixed effects. Subject identity was included as a random effect to account for flat group differences and increased RT time induced by task difficulty. F-contrasts were used to assess the variability explained by each one of the fixed effects for each linear mixed model. To assess statistical significance, and assuming that the peaks we selected as ROIs represent independent signals, we applied Bonferroni correction, correcting for the number of ROIs tested both in the task-specific (12 ROIs tested) and the group-specific regions (7 ROIs tested).

## 3 Results

### 3.1 Behavior

Table 1 presents the results of the behavioral analysis. As expected, accuracy was significantly higher for congruent than incongruent trials and for controls compared to patients, while mean RT and ex-Gaussian mu were significantly increased in the incongruent versus congruent trials and in patients compared to controls. The absolute difference in these measures between incongruent and congruent trials was significantly larger for patients compared to controls (significant task by group interaction) confirming a larger congruency effect. All RT-ISV measures except for RTCV (RTSD, ex-Gaussian sigma and tau) were not significantly different for incongruent versus congruent trials but were significantly larger for patients compared to controls. There was also no significant task by group interaction for these measures. The effect of congruency on RTCV was inverse to the effect observed for mean RT, namely RTCV was significantly larger for congruent compared to incongruent trials. RTCV was also significantly larger for patients compared to controls reflecting the effect of group on RTSD. Finally, there was a significant interaction of task by group interaction for RTCV that was again inverse to the interaction effect observed for mean RT, namely the increase in RTCV for congruent versus incongruent trials was significantly larger for controls compared to patients.

**Table 1:** **Behavioral measures of performance:** Means of all behavioral measures and standard errors of the means in parentheses. Results of the ANOVA analysis for task effect (congruent versus incongruent trials) group effect (patients versus controls) and task by group interaction. All RT measures are for correct trials only. RTM: Mean Reaction Time in ms, RTSD: Standard Deviation of RT in ms, RTCV: Coefficient of Variation of RT (ratio no units), mu: Mean of the Gaussian component of the ex-Gaussian distribution of RT in ms, sigma: Standard deviation of the Gaussian component of the ex-Gaussian distribution of RT distribution in ms, tau: mean of the exponential component of ex-Gaussian distribution of RT in ms. Significant values with p < .05 are marked in bold.

	Controls		Patients		Task (F_1_, p, η^2^)	Group (F_1_, p, η^2^)	Interaction (F_1_, p, η^2^)
	Congruent	Incongruent	Congruent	Incongruent			
Accuracy	99(0.7)	95.5(1.5)	97.8(0.7)	89(1.5)	63.5, **<10**^**−4**^,0.53	7.0, **0.01**,0.11	11.3, **0.001**,0.16
RTM	411 (11)	492(12)	478(11)	577(12)	585.9, **<10**^**−4**^,0.91	22.7, **<10**^**−4**^,0.28	6.7, **0.01**,0.10
RTSD	51.7(3.7)	57.9(3.1)	81.5(3.8)	81.8(3.1)	3.0, 0.09,0.05	35.5, **<10**^**−4**^,0.38	2.6, 0.11,0.04
RTCV	0.123 (0.006)	0.117 (0.004)	0.169 (0.006)	0.141 (0.004)	22.9, **<10**^**−4**^,0.29	27.1, **<10**^**−4**^,0.32	7.9, **0.007**,0.12
mu	372 (9)	447(12)	416(9)	519(12)	442.5, **<10**^**−4**^,0.86	16.6, **<10**^**−4**^,0.22	11.6, **0.001,**0.17
sigma	22.5(2.2)	23.8(2.9)	37.1(2.3)	42.1(2.9)	2.8, 0.10,0.05	26.8, **<10**^**−4**^,0.32	1.0, 0.32,0.02
tau	38.7 (4.2)	45.4 (3.8)	61.9 (4.3)	58.9 (3.9)	0.4, 0.35,0.01	14.2, **0.0004**,0.2	2.4, 0.12,0.04

Table 2 presents the resulting r^2^ values of the correlation of all behavioral measures with psychosis duration and dose of antipsychotic medication. These values represent the proportion of variance for each behavioral measure that can be explained by the corresponding clinical variable. As can be seen in Table 2, all of these correlations were small and none of them reached significance at *p* < .05.

**Table 2:** **Correlation of behavior to clinical variables**: Square of the Pearson correlation coefficient (r^2^) correlating each one of the behavioral measures of performance to the duration of psychosis and the dose antipsychotic medication expressed in chloropromazine equivalents. RTM: Mean Reaction Time, RTSD: Standard Deviation of RT, RTCV: Coefficient of Variation of RT, mu: Mean of the Gaussian component of the ex-Gaussian distribution of RT, sigma: Standard deviation of the Gaussian component of the ex-Gaussian distribution of RT distribution, tau: mean of the exponential component of ex-Gaussian distribution of RT.

	Duration (r^2^)	Medication (r^2^)
Accuracy congruent	<0.01	<0.01
Accuracy incongruent	0.05	<0.01
RTM congruent	0.04	<0.01
RTM incongruent	0.00	0.06
RTSD congruent	0.08	0.01
RTSD incongruent	0.06	0.05
RTCV congruent	0.08	<0.01
RTCV incongruent	0.06	<0.01
Mu congruent	0.01	<0.01
Mu incongruent	0.10	0.06
Sigma congruent	0.01	<0.01
Sigma incongruent	0.09	0.03
Tau congruent	0.03	0.07
Tau incongruent	0.12	<0.01

### 3.2 Whole-brain analysis

**Fig. 1:**
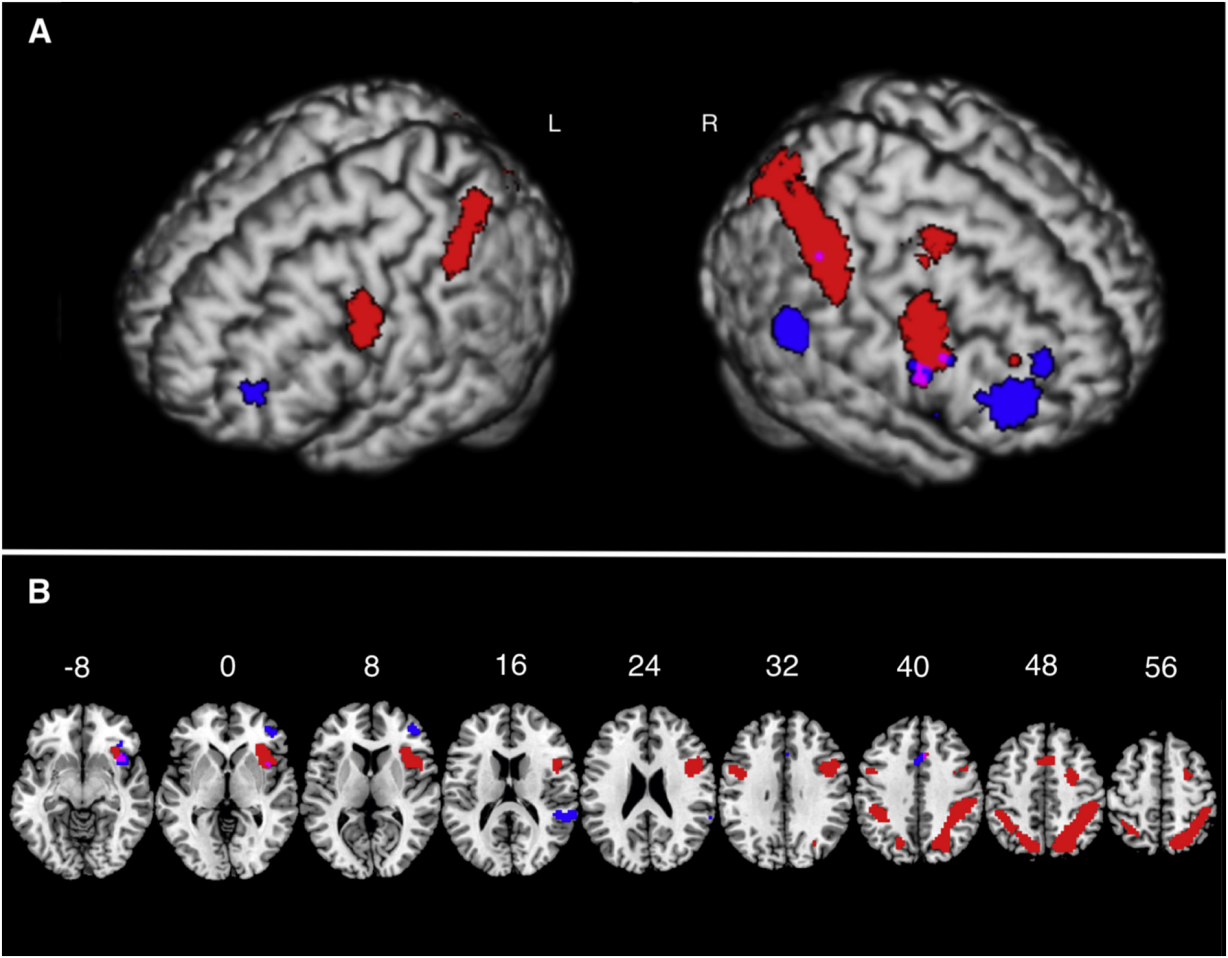
**Whole brain analysis:** (A) Maximum intensity projection rendering of the results of the group level whole-brain analysis. Red – Task effects (Incongruent > Congruent trials); Blue – Group effects (Controls >. Patients contrast). (B) Multi-slice view of the same results. A small overlap (magenta) can be observed between the two contrasts in the region of Insula R. Display threshold is p < .05 FWE. (For interpretation of the references to color in this figure legend, the reader is referred to the web version of this article.)

A t-contrast was used to assess the effect of trial type (congruent versus incongruent trials). Clusters that were significantly more activated for incongruent versus congruent trials are presented in Fig. 1 (red color) and Table 3. There was no significant interaction between trial type and group for these clusters. There were no significant effects for the opposite contrast (congruent trials > incongruent trials). A t-contrast was also used to assess the effect of group. The clusters that were significantly more activated in the control group versus the patient group are shown in Fig. 1 (blue color) and Table 4. There was no significant interaction between trial type and group for these clusters and there were no significant effects for the opposite contrast (patients > controls).

**Table 3:** **Effect of task on brain activation:** This table presents the areas that were significantly more activated for Incongruent versus Congruent trials (t-contrast). Only correct responses are included in this analysis. Peak-level significance *p* < .05 corrected using Family Wise Error (FWE) correction. Only clusters significant at *p* < .05 FWE are included. Anatomic labeling was made using the aal atlas.

Anatomical label	Cluster size	p-FWE	Peak T	Peak p-FWE	x	y	z
Bilateral parietal lobes							
Inferior parietal R	681	<.001	6.95	<.001	42	−37	49
Inferior parietal R – 2			6.53	<.001	36	−46	52
Postcentral R			6.82	<.001	45	−31	43
Inferior parietal L	293	<.001	5.72	.001	−42	−40	43
Superior parietal L			5.14	.009	−18	−70	43
Precuneus L			5.29	.004	−12	−73	49

Bilateral frontal lobes							
Precentral R	441	<.001	6.52	<.001	48	8	31
Inferior frontal opercular R			5.95	<.001	45	8	7
Insula R			6.51	<.001	36	23	1
Supplementary frontal L	68	<.001	5.77	<.001	27	−1	46
Precentral L	75	<.001	4.21	<.001	−45	2	34
Supplementary motor L	56	.001	3.40	<.001	3	14	49

**Table 4:** **Effect of group on brain activation:** This table presents the areas that were significantly more activated in the control group versus the patient group (t-contrast). Only correct responses are included in this analysis. Peak-level significance *p* < .05 corrected using Family Wise Error (FWE) correction. Anatomic labeling was made using the aal atlas. Only clusters significant at *p* < .05 FWE are included.

Anatomical label	Cluster size	p-FWE	Peak T	Peak p-FWE	x	y	z
Right frontal lobe							
Middle frontal R	167	<.001	5.67	<.001	45	44	7
Inferior frontal orbital R			5.07	<.001	36	26	7
Insula R			5.45	<.001	42	11	−2

Right temporal lobe							
Superior temporal R	78	<.001	6.19	<.001	63	−40	19
Superior temporal R – 2			5.96	<.001	51	−40	16

Cingulate							
Middle cingulate L	56	<.001	5.22	<.001	0	14	37
Anterior cingulate R			4.72	<.001	3	23	22

In order to further examine a possible interaction effect between these two factors we also took the incongruent > congruent trial first-level T-contrast statistical maps for each subject as an input to the group level analysis and specified a two-sample T-contrast design, since T-contrasts testing the effect of one condition against the other are more sensitive than those testing the effect of a condition against baseline. However, this design did not reveal any interaction effect between group and trial type either. Finally, medication (chlorpromazine equivalents) and duration of psychosis had no effect on the results when used as covariates.

### 3.3 ROI analysis

**Fig. 2:**
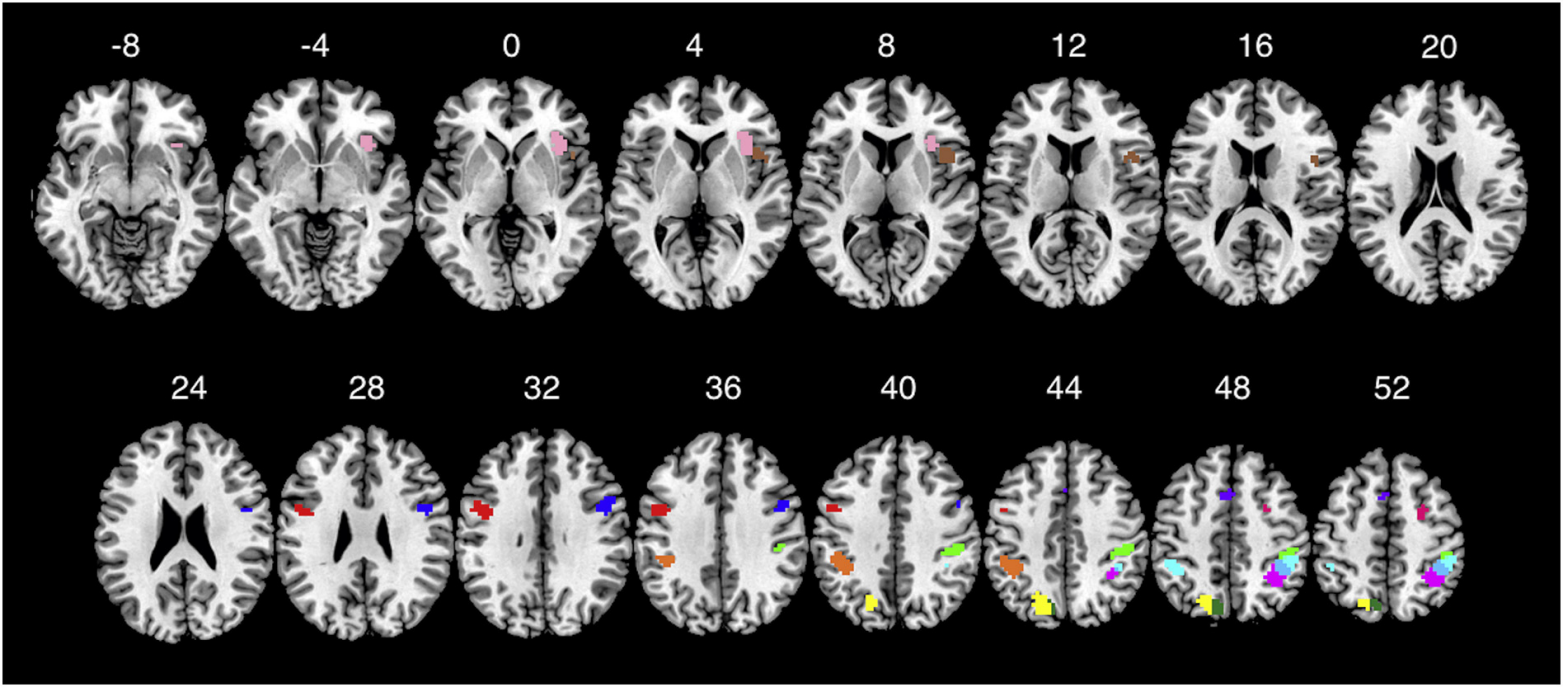
**Task related ROIs:** Multi-slice view of group anatomical ROIs constructed from all (twelve) significant peaks from the task contrast as described in Table 2, color-coded to be easily discriminated. Cyan – Inferior Parietal R, Green – Postcentral R, Magenta – Inferior Parietal R 2, Blue – Precentral R, Pink – Insula, Brown – Inferior Frontal Opercular R, Fuchsia – Supplementary Frontal R, Orange – Inferior Parietal L, Yellow – Precuneus L, Dark green – Superior Parietal L, Blue – Precentral L, Purple – Supplementary Motor L. Display threshold is 0.05 FWE. (For interpretation of the references to color in this figure legend, the reader is referred to the web version of this article.)

Correlation coefficient estimates for the linear mixed model using trial-type specific ROI activations (Fig. 2) to predict RT measures are presented in Table 5. Statistically significant correlation coefficients are marked in bold. Significant effects were only observed for tau. All coefficients were negative, suggesting that decreased activation in these areas correlated with an increase in RT-ISV as measured by tau. In the same linear model, the ROI-by-group interaction was not significant for any of these task-related ROIs, suggesting that the correlation of activation to tau was not different between patients and controls. In contrast there was a significant ROI-by-trial-type interaction for the ROI activation centered at the peak in postcentral right area (beta = 0.74, *p* < .001), in which case there was a negative slope for the congruent trials and a non-significant slope for incongruent trials. Thus in this area the decrease in tau with increasing ROI activation was specific for congruent trials but not incongruent trials. Another significant ROI-by-trial-type interaction was observed for the ROI activation centered at the peak in the inferior frontal opercular right area (beta = 0.34, *p* = .036), in which case both trial-type slopes were negative and statistically significant.

**Table 5:** **Relation of trial type specific ROIs to RT measures:** This table presents the beta coefficients for all mixed models predicting each one of the behavioral measures of RT (RTM: Mean RT, RTSD: Standard deviation of RT, mu, sigma and tau of the ex-Gaussian distribution of RT) with the ROI effect size for all trial-type specific ROIs. All betas that were significant at *p* < .05 are marked in bold.

	RTM	RTSD	RTCV	Mu	Sigma	Tau
Inferior parietal R	−0.03	−0.07	−0.02	−0.05	0.08	−0.21
Inferior parietal R – 2	−0.06	−0.19	−0.03	−0.01	0.13	−0.42
Postcentral R	−0.0.6	−0.14	−0.12	−0.04	0.05	**−0.48**
Inferior parietal L	−0.07	−0.08	−0.06	−0.07	−0.03	−0.16
Superior parietal L	−0.06	0.02	0.08	−0.06	0.06	−0.07
Precuneus L	−0.13	−0.15	0.01	−0.07	−0.05	−0.27
Precentral R	0.13	0.09	−0.14	−0.04	−0.15	0.11
Inferior frontal opercular R	0.03	−0.20	0.02	0.01	0.01	**−0.44**
Insula R	0.13	−0.15	0.55	0.12	−0.10	−0.45
Supplementary frontal L	0.21	−0.23	0.11	0.16	0.11	**−0.45**
Precentral L	0.04	−0.14	−0.04	−0.14	−0.10	−0.20
Supplementary motor L	0.17	−0.23	0.06	0.11	−0.05	**−0.45**

**Fig. 3:**
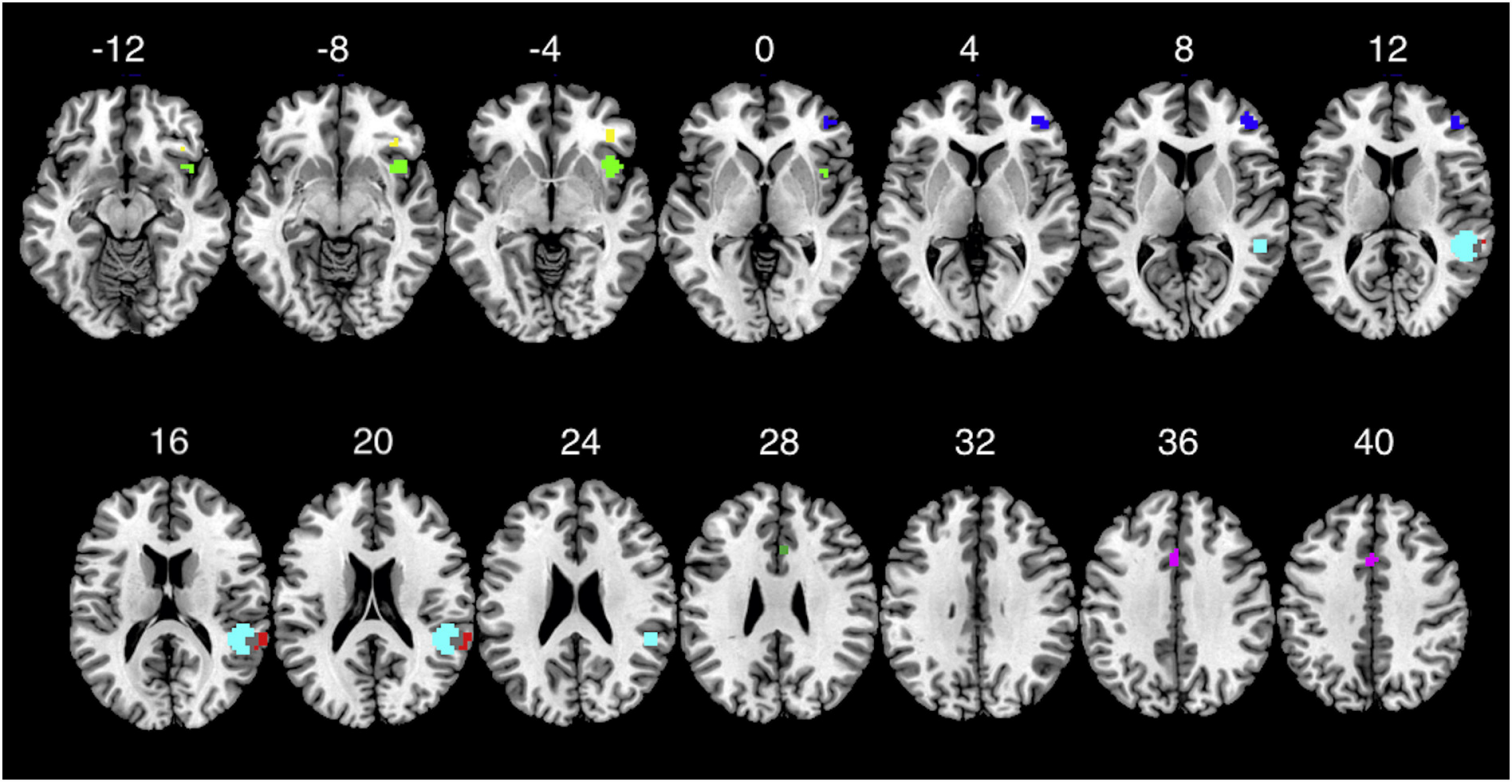
**Group related ROIs:** Multi-slice view of group anatomical ROIs constructed from all (seven) significant peaks from the group contrast as described in Table 1, color-coded to be easily discriminated. Red – Superior Temporal R, Cyan – Superior Temporal R 2, Blue – Middle Frontal R, Green – Insula R, Yellow – Inferior Frontal Orbital R, Magenta – Middle Cingulate L, Purple – Anterior Cingulate R. Display threshold is 0.05 FWE. (For interpretation of the references to color in this figure legend, the reader is referred to the web version of this article.)

Table 6 shows correlation coefficient estimates for the linear mixed models using group-specific ROI activations (Fig. 3) to predict RT measures. The statistically significant correlation coefficients are marked in bold. Again, significant correlations were observed only for RT-ISV measures. All coefficients were negative, indicating that a decrease in activation at these ROIs resulted in an increase in RT-ISV measures. The correlation of insula right ROI to RTCV was significantly modulated by trial type (trial-type interaction, beta = 0.45 *p* = .017) but not by group (beta = 0.09, *p* = .68). The correlation of frontal middle right ROI to RTCV was also significantly modulated by trial type (trial-type interaction, beta = 0.31 *p* = .035) but not by group (beta = 0.31, *p* = .138). Finally, the correlation of middle cingulate left ROI to tau was modulated by trial type (trial-type interaction, beta = 0.70 *p* = .001) but not by group (group interaction, beta = 0.31 *p* = .22). These task-specific effects stem from the fact that the decrease in activation in these ROIs correlated with an increase in RTCV and tau much stronger for congruent trials than for incongruent trials.

**Table 6:** **Relation of group specific ROIs to RT measures:** This table presents the beta coefficients for all mixed models predicting each one of the behavioral measures of RT (RTM: Mean RT, RTSD: Standard deviation of RT, mu, sigma and tau of the ex-Gaussian distribution of RT) with the ROI effect size for all group specific ROIs. All betas that were significant at *p* < .05 are marked in bold.

	RTM	RTSD	RTCV	Mu	Sigma	Tau
Middle frontal R	−0.13	**−0.39**	**−0.43**	−0.04	**−0.35**	**−0.69**
Inferior frontal orbital R	0.17	0.06	−0.06	0.01	−0.15	−0.12
Insula R	0.15	−0.25	**−0.49**	0.16	−0.26	**−0.59**
Superior temporal R	−0.15	−0.26	−0.23	−0.06	−0.23	−0.38
Superior temporal R – 2	−0.18	−0.28	−0.18	−0.13	−0.20	−0.35
Middle cingulate L	0.05	**−0.43**	**−0.56**	0.05	−0.22	**−0.56**
Anterior cingulate R	0.06	−0.19	−0.36	0.05	−0.17	−0.33

**Fig. 4:**
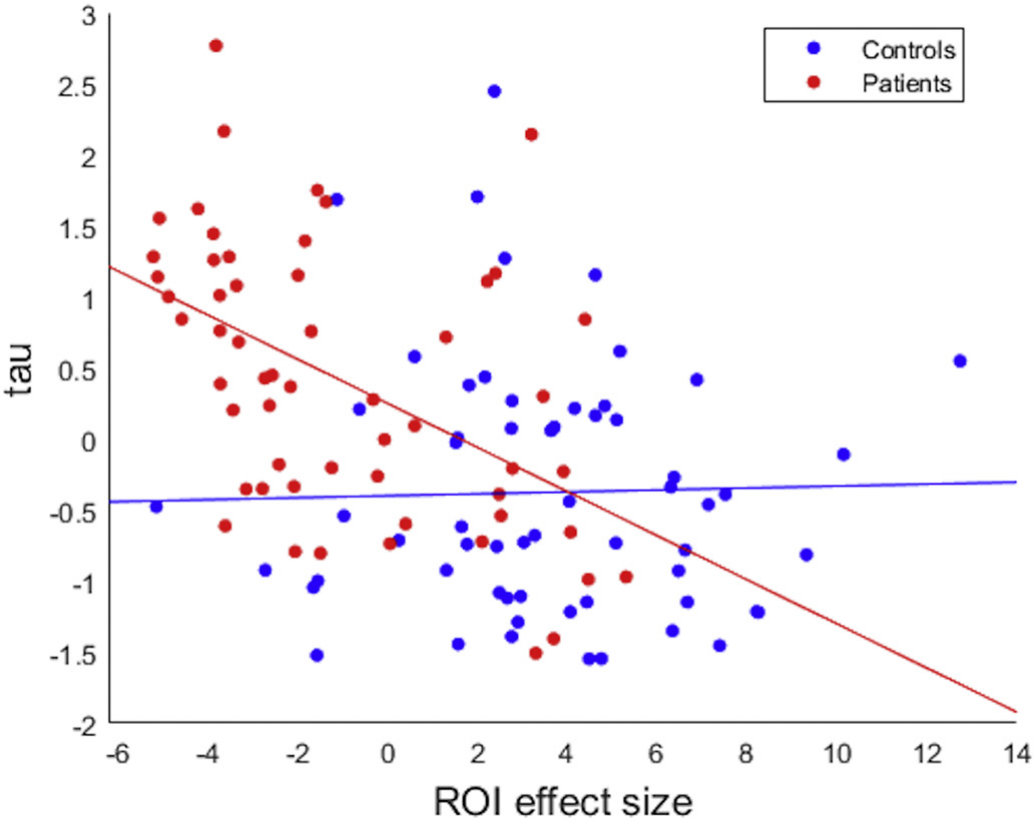
**ROI to tau relation:** ROI effect size (y-axis) on tau (x-axis) for Middle Frontal R estimated across conditions for each group separately, using the linear mixed model. Fixed effects are reported and plotted. Controls: *r* = 0.007, *p* = .826, Patients: *r* = −0.16, *p* < 10^−4^).

The correlation of middle frontal right ROI activation to tau was the only one that was modulated by group (group interaction, beta = 0.58 *p* = .014). This correlation was not modulated by trial type (trial-type interaction, beta = 0.25 *p* = .16). As seen in Fig. 4, the decrease in activation in this ROI resulted in increased tau for the patient group but not for the control group. A linear mixed model was estimated separately for each group. Since there was no interaction with trial type this estimation was performed on the concatenated data from both trial types. This analysis confirmed that the significant negative correlation of ROI activation and tau was observed only for patients (beta = −0.16, *p* < 10^−4^) but not for controls (beta = 0.007, *p* = .863).

## 4 Discussion

In this study, the Eriksen flanker spatial attention task was used to investigate RT distributions and their relation to functional brain activation in patients suffering from schizophrenia and in healthy controls matched for age and sex.

Patients with schizophrenia produced more errors in their performance compared to healthy controls, while both groups produced significantly more errors in the incongruent trial type compared to the congruent (congruency effect). Moreover, the congruency effect on error rate was significantly larger for patients compared to controls. This result validated the use of the Eriksen flanker task in this study for measuring attention and confirmed a deficit in attention control for patients. The same deficit has been observed in previous studies using both the Eriksen flanker task (Gooding et al., 2006; Wang et al., 2005), as well as other tasks where executive control of attention was manipulated (Heathcote et al., 2015). The mean RT was larger overall in the patient group and it was larger for incongruent compared to congruent trials for both groups. This finding also provides validation for the use of the particular task in the current study showing that mean RT was sensitive to the attentional load of the task (congruency effect). Furthermore, this congruency effect was larger for patients compared to controls confirming a deficit in the control of attention for patients. This deficit was in line with findings from previous studies using the Eriksen flanker task (Gooding et al., 2006; Wang et al., 2005), as well as other tasks of executive control of attention in schizophrenia (Bunge et al., 2002; Heathcote et al., 2015; Yarkoni et al., 2009). Thus, at the behavioral level we confirmed a deficit in the control of attention for patients with schizophrenia expressed in both performance accuracy and performance speed.

The focus of the current study was on RT-ISV measures of performance. All these measures (RTSD, RTCV, sigma and tau) were significantly larger in patients compared to controls confirming results of previous studies measuring RT-ISV in schizophrenia (Fassbender et al., 2014; Fish et al., 2018; Kaiser et al., 2008; Karantinos et al., 2014; Rentrop et al., 2010). All but one RT-ISV measures (RTSD, sigma and tau) were not related to the attentional manipulation in the Eriksen flanker task since we did not observe a significant congruency effect for these measures in neither patients nor controls. Furthermore, there was no interaction between congruency and group for these measures. These results provide strong evidence for the dissociation of measures of RT-ISV and mean RT as well as performance accuracy in this task and suggest that RT-ISV assesses different parameters of behavior. Moreover these results do not support the hypothesis that RT-ISV is related to attention control. A separate notion should be made here for RTCV. This measure was significantly related to congruency and it was observed that RTCV was larger for congruent compared to incongruent trials. There was also an interaction of congruency with group and the congruency effect was larger for controls compared to patients. These results are exactly the reverse from those observed for the mean RT. This would be expected if these effects were only related to the mean RT and there were no effects on RTSD. Then the reverse effects would be observed for 1/mean RT. RTCV was also significantly larger for patients compared to controls and this would be expected if the difference in RTSD between the two groups would be much larger than the difference in mean RT. Again, this is true because the increase in RTSD for the patient group versus the control group was much larger (49.1%) compared to the increase in mean RT (16.8%). These results suggest that RTCV is highly sensitive to the effects of mean RT and is not a clear measure of RT-ISV.

In conclusion the behavioral results of this study showed a clear dissociation of RT-ISV measures of performance in the Eriksen flanker task from both performance accuracy and the mean response speed (mean RT). RT-ISV was increased for patients compared to controls and this effect was not related to attention. One previous study used an attentional task (the Stroop task) to investigate RT-ISV in patients with schizophrenia and healthy controls. The authors showed that RT-ISV measures (sigma and tau) increased for patients in both incongruent and congruent trial types (Fassbender et al., 2014). Although in that study a significant congruency effect was observed for RT-ISV this congruency effect was the same for patients and controls (no interaction of congruency and group). These results together with the results of the current study suggest that differences in RT-ISV between patients and controls are not linked to a deficit in the control of attention.

The whole brain analysis of fMRI data confirmed that a number of different brain areas were more active in the incongruent versus congruent trials for all participants (congruency effect) while there were no areas that were more activate for congruent versus incongruent trials. This task-related set of brain areas included prefrontal and parietal cortical areas in both hemispheres, as well as the right insula and the left supplementary motor cortex. Previous fMRI studies using the same task also found activation in areas of the prefrontal and parietal cortex (Bunge et al., 2002).

The task-related set of areas was similarly activated in both patients and controls and there was no difference in activation that might correlate with the difference in the effect of congruency on accuracy and speed of performance between the two groups. A previous study using the same task in patients with schizophrenia and healthy controls (Voegler et al., 2016) focused only in the activation of the middle cingulate cortex during the commission of errors and found reduced activation for patients. The current study reports all areas that were significantly activated by the congruency effect and showed no difference in activation of these areas between patients and controls.

The whole-brain analysis of the fMRI data also revealed differences in activation in specific brain areas that were more activate in controls compared to patients. The opposite effect, namely increased activation for patients compared to controls was not observed. This group-related set of areas included the right prefrontal cortex, the right superior temporal cortex, the middle and anterior cingulate cortex and the right insula (with a different peak than that observed in the task specific activation of the same area). Interestingly, there was no effect of congruency in these areas and if one compares the two sets of task-related and group-related areas there is no overlap (see Fig. 2, Fig. 3).

Our main focus in the current study was to investigate the relation of brain activation to measures of RT-ISV and more specifically if this relation could explain the differences in these measures between patients with schizophrenia and healthy controls. Using ROIs from the task-related areas we observed significant negative correlations between activation in some of these areas (including the right prefrontal cortex, the right insula and the left supplementary frontal and supplementary motor cortex) and tau which is a measure of the non-Gaussian RT-ISV. The increase in activation corresponded to a smaller tau. Could this correlation also explain the difference in tau between patients and controls? The behavioral analysis as well as the whole brain analysis of fMRI data had already suggested that the effects of attention were confined to the mean RT and accuracy with no effect on RT-ISV measures. Moreover, differences in RT-ISV measures between patients and controls were not related to the effect of attention. Finally, these brain areas that were related to attention were not differentially activated between patients and controls. Based on these results our hypothesis was that the correlation of tau with ROI activation in the task related areas would not be different between patients and controls and this is what was observed.

Using ROIs from the group-related set also resulted in negative correlations of ROI activations with measures of RT-ISV in areas including the Right Middle Frontal, Right Superior Temporal and Left Middle Cingulate. Interestingly the ROI activation for the Right Middle Frontal area was negatively correlated with all measures of RT-ISV as seen in Table 6. Could these correlations explain the difference in RT-ISV between patients and controls? Based on our previous results we expected that ROI activations in some of these areas that were less activated in patients compared to controls would predict the differences in RT-ISV measures between the two groups. Indeed, the decrease in the ROI activation of the right middle frontal area in patients correlated strongly with the increase in RT-ISV as measured with the ex-Gaussian tau. This finding provides strong support for the hypothesis that RT-ISV and especially the fat tail of the RT distribution as modelled by the ex-Gaussian tau is a specific measure of cognitive and sensorimotor processing that is independent of the mean RT. Furthermore the fact that the effect of RT-ISV measures on brain activation was observed in areas that were not related to the modulation of attention is also in favor of the dissociation between mean RT and RT-ISV and further suggests that the increase in RT-ISV in schizophrenia is not related to a failure in the control of attention. What is the cognitive process then that is related to the activation of the middle frontal cortex and its impairment due to hypo-activation of this area manifests as increased RT-ISV in schizophrenia?

Activation of the bilateral dorsolateral prefrontal cortex was found to be specifically correlated with RT-ISV in a go-no/go task in healthy adults (Bellgrove et al., 2004). In that study authors first identified the areas that were more active in the no-go compared to the go trials and then observed in which of these areas the magnitude of activation correlated significantly with RTCV. The correlation of the magnitude of activation in the dorsolateral prefrontal cortex and RT-ISV was positive in that study suggesting a link between increased activation at these areas and increased RT-ISV. The authors concluded that dorsolateral prefrontal cortex is activated during inhibitory control. In turn, higher activation of these areas in individuals with higher RT-ISV reflects a greater requirement of top-down inhibitory control.

We could thus hypothesize that the patients' failure to activate areas specifically related to the control of inhibition, such as the dorsolateral prefrontal cortex could result in larger RT-ISV. There is a large literature providing evidence that hypo-activity in the dorsolateral prefrontal cortex is present in patients with schizophrenia and this hypo-activity has been related to deficits in inhibitory control of behavior (Lesh et al., 2013; Minzenberg et al., 2009; Yoon et al., 2008). It is therefore suggested that this hypo-activity could also result in larger RT-ISV and loss of cognitive stability in schizophrenia. Further support for this hypothesis comes from a study which tested the effects of lesions in different prefrontal brain areas on RT-ISV (Stuss et al., 2003). Findings showed that patients with lesions specifically in the left and right dorsolateral prefrontal cortex had increased RT-ISV in a series of fast decision tasks including a simple RT task, as well as simple and complex two-choice RT tasks.

## 5 Conclusions

This study confirmed that patients with schizophrenia show a very large increase in RT-ISV in the spatial version of the Eriksen flanker task. This increase was not modulated by congruency, suggesting that it is not related to a deficit in the control of spatial attention in these patients. Similarly, the set of brain areas whose activation reflected the congruency effect in this task (increased activation during incongruent condition) was unrelated to the RT-ISV differences between patients and controls. On the other hand, hypo-activation in an area of the right dorsolateral prefrontal cortex in patients correlated specifically with decreased RT-ISV. Hypo-activity of dorsolateral prefrontal cortex has been linked to a deficit in inhibitory control of action in schizophrenia. Therefore, it is possible that a common neural substrate mediates the deficit of inhibitory cognitive control and the increase in RT-ISV in these patients.
